# Medical Items

**Published:** 1896-08

**Authors:** 


### MEDICAL ITEMS.

Dr. J. B. S. Holmes has been appointed a member of the State Medical Examining Board by the Governor, to succeed Dr. J. C. Olmsted, resigned.

Dr. L. B. Grandy is spending several months in the schools of New York fitting himself for special practice in diseases of the Eye, Ear, Nose, and Throat. He will return to Atlanta some time in the fall.

At the last meeting of the Anatomical Board of Georgia, Dr. Wm. Perrin Nicolson was elected President. Upon the resignation of Dr. L. B. Grandy, Secretary and Treasurer, Dr. J. L. Campbell was elected to fill this position.

Dr. Caverhill, medical officer of health, Haddingtonshire County Council, found that by the use of Pasteur filters, giving 1,000 gallons per day of sterilized water in connection with certain wells in the county, he was apparently able to limit the spread of typhoid fever in the district. During an epidemic none of the persons using such water were attacked.

Ammonol, so the manufacturerers and proprietors say, is "Ammoniated Phenylacetamide ($C_6H_5NH_2$)." Dr. R. G. Eccles (*Druggists' Circular*, May, 1896) has called attention to the fact that this chemical name is only a subterfuge on the part of the proprietors. Phenylacetamide is only another name for acetanilid, and the formula $C_6H_5NH_2$ is really that of an anilin oil. Dr. Eccles says that all the medicinal results from ammonol may be got from a mixture of six parts acetinalid, three parts sodium bicarbonate, and one and a half parts ammonium carbonate. The yellowish tinge is obtained from the addition of a little methyl orange and curcumin.

MISSISSIPPI VALLEY MEDICAL ASSOCIATION—MEETING AT ST. PAUL, MINN.—We desire to draw the attention of our readers to the fact that the meeting of this society has been changed to September 15, 16, 17, 18, and that plans are being formed, if 100 members can be got together, to have a trip to the Yellowstone Park. Particulars will be supplied by Dr. C. A. Wheaton, St. Paul, Minn.

---

THE following is the announcement of the new *Western Medical Review:* A medical journal for the progressive physician. A Western medical monthly magazine for the Western doctor. First-class, clean, and in every way up to date. Nothing cheap about it but the price. That is only two dollars per year. We claim quality and not quantity, but for all that the *Western Medical Review* will contain more original reading matter than any other first-class two-dollar medical journal published. It is a paper for you to patronize, and we want your patronage. Can we have it? Address WESTERN MEDICAL REVIEW, Lincoln, Neb.

---

DR. JOSEPH WEATHESSLY, of Waco, Miss., sends us the results of his experience in the treatment of typhoid fever in the last forty years. In a recent epidemic he treated sixty-three cases, with a mortality of three, and these died from causes unconnected with the fever. For constipation he uses sulphate of magnesia; chlorate of potash "for the soreness and ulceration of the bowels," with ten drops of spirits of turpentine; the patient is sponged with lukewarm water every six hours for the fever. For rest he prefers the sulphate of morphia "*ad libitum*" (though most physicians will question the wisdom of this step); nux vomica for delirium; he has also lately used arsenite of potash.

---

THE MISSISSIPPI VALLEY MEDICAL ASSOCIATION.—A meeting of the Executive Committee of the Mississippi Valley Medical Association was held at Atlanta on May 6th, and the following gentlemen were appointed to deliver addresses: Dr. H. N. Moyer, Chicago, Address on Medicine; Dr. Horace H. Grant, Louisville, Address on Surgery. The indications are that the meeting to be held at St. Paul, on October 20, 21, 22, and 23, will be the largest and most successful in the history of the Association. As all the railroads will offer reduced rates for the round trip, an opportunity will be given to visit St. Paul and Minnesota during the most delightful season of the year. H. O. Walker, M.D., Detroit, Mich., President; C. A. Wheaton, M.D., St. Paul, Minn., Chairman Committee of Arrangements; H. W. Loeb, M.D., 3559 Olive street, St. Louis, Secretary.

---

"THE LARYNGOSCOPE."—We have received the first number of this new monthly journal, devoted to diseases of nose, throat, and ear, and intended "to fill the niche between the strictly special and the general journals with that class of physicians who confine themselves entirely to the treatment of the diseases mentioned, or who pay special attention to these troubles while maintaining a general practice." The editors are Drs. Frank Y. Rumbold and M. A. Goldstein, St. Louis, and the associate editors are Drs. Lederman, New York; Scheppegrell, New Orleans; Bishop, Chicago; Eaton, San José, Cal.; while the journal is fortunate enough to have for its list of foreign editors, Drs. Durdas Grant, London; Kirk Duncannon, Edinburgh; Kimaru, Tokio; Urbantschitsch, Vienna; Hartmann, Berlin; and Sterling Ryerson, Toronto. The subscription is $2.00 a year. If this journal continues as it has begun, we venture to prophecy for it a successful career.

---

THE following experience of Slatin Pasha, recorded in his "Fire and Sword in the Sudan," p. 70, should help to reconcile patients to the comparatively mild unpleasantnesses of more civilized professors of the healing art: "Without a moment's hesitation the doctor placed his hand on my head, pressed my temple with his thumb and forefinger, and, muttering a few words I could not understand, to my horror spat in my face. In a moment I had jumped up and knocked him down; but Ahmed, who was standing by leaning on his crutch, begged me not to take it in this way. 'It was not really meant for rudeness,' he said; 'it is merely a part of the cure, and will do you much good.' But the poor doctor, whose confidence had been somewhat shaken and was still standing at a distance, muttered, 'Headache is the work of the devil, and I must drive it out; several passages from the Koran and the sayings of holy men direct that it should be chased away by spitting, and thus his evil work in your head will cease!'"

---

WE heartily commend the following abstract from a paper read by Professor J. Bradford McConnell before the Montreal Medico-Chirurgical Society, May 29, 1896, and published in the *Canada Medical Record.* Too much care cannot be observed in the protection of milk supplies from the time the milk leaves the cow until its use, which we think should, specially in the case of children, always be in the cooked state.

The adoption of a minimum standard would result in the sale of a very large quantity of adulterated milk, which, apart from diminishing the nutritive value of the milk—a matter of great importance in infant-feeding—is often the immediate cause of transmitting disease germs by the addition of infected water. In the opinion of the writers all this can be prevented and milk of uniform standard can be obtained by encouraging the establishment of milk depots like the Walker-Gordon laboratory of Boston. In the management of this establishment farm and herd are under the absolute control of the laboratory, and are used for laboratory purposes only; the cows, their food, their stables, their pasture, and their drinking water are subjected to the frequent, paid, critical examination of the best veterinary surgeon that can be procured in Boston. The dairymen dress in white suits before milking, each having previously had a bath. The cows are milked into glass pails, and the milk, after being aerated and cooled to about 44 degrees Fahrenheit in a tank of ice and water, is delivered at the laboratory in Boston within four hours of the milking.

At the laboratory a ventilating engine keeps up a constant change of air, and a hose keeps the enameled brick walls and stone floors wet to prevent any remaining dust from contaminating the milk while it is being "modified." The whole milk, after being "Pasteurized," passes through a Stockholm separator which makes sixty-eight hundred revolutions a minute, yielding a cream of an almost constant 16 per cent. fat. It not only does this, but it removes all dirt that from unavoidable causes has gained access to the milk, thus yielding a clean, skimmed milk, practically free from fat (only 0.13 per cent. remaining).

